# Complementary medicines used in ulcerative colitis and unintended interactions with cytochrome P450-dependent drug-metabolizing enzymes

**DOI:** 10.55730/1300-0144.5482

**Published:** 2022-07-28

**Authors:** Alaattin ŞEN

**Affiliations:** 1 Department of Molecular Biology and Genetics, Faculty of Life and Natural Sciences, Abdullah Gül University, Kayseri, Turkey; 2 Department of Biology, Faculty of Arts and Sciences, Pamukkale University, Denizli, Turkey

**Keywords:** Cytochrome P450, CYPs, ulcerative colitis, complementary medicines, herbal products, adverse effect

## Abstract

Ulcerative colitis (UC) is an idiopathic, chronic inflammatory disease with multiple genetic and a variety of environmental risk factors. Although current drugs significantly aid in controlling the disease, many people have led to the application of complementary therapies due to the common belief that they are natural and safe, as well as due to the consideration of the side effect of current drugs. Curcumin, cannabinoids, wheatgrass, Boswellia, wormwood and Aloe vera are among the most commonly used complementary medicines in UC. However, these treatments may have adverse and toxic effects due to unintended interactions with drugs or drug-metabolizing enzymes such as cytochrome P450s; thus, being ignorant of these interactions might cause deleterious effects with severe consequences. In addition, the lack of complete and controlled long-term studies with the use of these complementary medicines regarding drug metabolism pose additional risk and unsafety. Thus, this review aims to give an overview of the potential interactions of drug-metabolizing enzymes with the complementary botanical medicines used in UC, drawing attention to possible adverse effects.

## 1. Introduction

Inflammatory bowel diseases (IBDs) are idiopathic inflammatory disorders that have a chronic course characterized by exacerbation and remission throughout an individual’s lifetime. It is thought that a genetic predisposition as well as environmental factors play a role in the manifestation of these diseases. IBDs are classified into two types: ulcerative colitis (UC) and Crohn’s disease (CD) [1, 2]. UC and CD are distinguished by the sections of the digestive system affected and by phases of relapses and remission, causing symptoms that are somewhat different between the two diseases [3–5]. UC is still an idiopathic inflammatory disease of the colonic mucosa with an unknown aetiology that is more prevalent globally than CD. Interaction between genetic and environmental factors, immune dysfunction, intestinal flora dysbiosis, and impaired intestinal mucosal barrier function are currently the leading causes of IBD. UC, with an accelerating global incidence, has an unfavourable influence on patients’ health as well as their emotional, social and occupational life [2].

Although the burden of the disease is increasing, therapy for UC is challenging due to the absence of clearly defined medical treatments to manage periods of recurrence and remission with long-term consequences. However, drugs such as 5-aminosalicylate, azathioprine, 6-mercaptopurine, cyclosporine, and antitumour necrosis factor monoclonal antibodies have been utilized to treat UC [6,7]. The main focus of UC treatment is to improve quality of life with minimum steroid exposure while also alleviating symptoms and mucosal inflammation. Annual direct medical costs per patient in the United States ranged from $6217 to $11,477 and from €8949 to €10,395 in Europe, posing a significant financial burden on patients, families, and governments [8–11]. Thus, the use of complementary and alternative medicine (CAM) among UC patients is popular, with herbal therapies being the most commonly utilized form of treatment. It is estimated that 28.9% of the US population use one or more natural medicines on a regular basis, with herbal products accounting for 9.6%–12.1% of these. Various herbal products used in the clinic have been found in studies to be useful in the treatment of UC [12–18].

**Figure 1:**
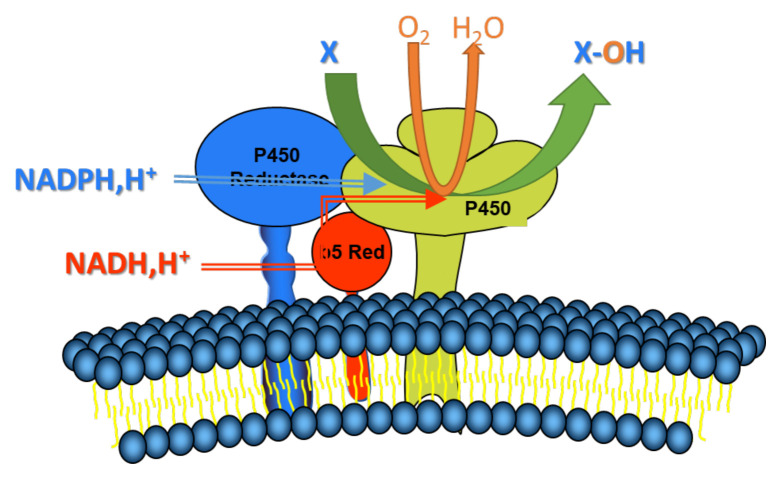
The microsomal electron transport system (P450Reductase: Cytochrome P450 Reductase; b5 Red: Cytochrome b5 Reductase; P450: Cytochrome P450 Reductase; X: Substrate).

The microsomal cytochrome P450 system is a product of the CYP superfamily of genes, and it is a key electron transport chain in the membrane of the endoplasmic reticulum of the cell. These enzymes are critical in the oxidative activation, inactivation, and excretion of the majority of xenobiotics and drugs, as well as in controlling the degree of their toxicity, among other functions [19–24]. Cytochrome P450 catalyses the oxidation of lipophilic compounds by introducing one atom of molecular oxygen into the substrate, transforming the latter into either less toxic or completely harmless hydrophilic metabolites that may be excreted from the body. The electron travels from NADPH or NADH via a flavoprotein cytochrome P450 reductase or a cytochrome b5 reductase to distinct isoforms of cytochrome P450 (CYP450s) during oxidative metabolism in the microsomal microenvironment involving the cytochrome P4S0 system (Figure 1) [25–27]. Many endobiotic and xenobiotic oxidation events are facilitated by cytochrome P450s, which are the terminal oxidase components of an electron transport system found in the endoplasmic reticulum and responsible for many of these reactions.

**Figure 2:**
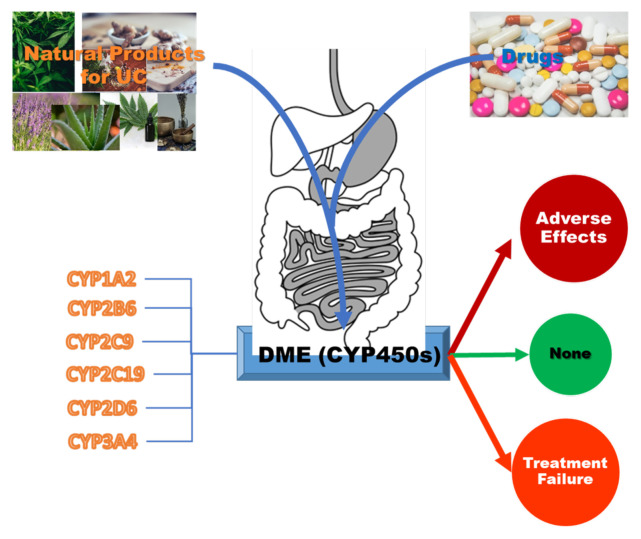
Interactions of drugs with natural products involving drug metabolising enzymes (DME).

Humans express a total of 57 CYP proteins; of these, seven are found in mitochondria, where they catalyse particular oxidation events associated with steroid metabolism. Only six genes —CYP1A2, CYP2C9, CYP2B6, CYP2C19, CYP2D6, and CYP3A4— make substantial contributions to drug clearance out of the remaining 50 genes studied. It has been estimated that approximately 60 % of all drug-related material provided to patients is cleared by these six CYPs. The CYP3A subfamily is responsible for metabolizing approximately half of the medicines that have been cleared by CYPs [28–30]. The role of some critical CYP isoforms in the pathogenesis or aetiology of ulcerative colitis concerns gene polymorphisms [31]. In addition, their significance in metabolism concerning ulcerative colitis in patients has been reported [32]. Plant-derived phytochemicals are also substrates for various subtypes of the CYP450 family and can induce or reduce the activity of these enzymes; thus, the concurrent use of phytochemicals and conventional drugs can cause an increase or decrease in serum levels of both substances via induction or inhibition of CYP450 activity (Figure 2).

A computerized search of PubMed, Web of Science, and Scopus for randomized controlled trials involving ulcerative colitis was conducted to identify the natural plants used by patients to treat ulcerative colitis. The unfavourable effects of these natural products on cytochrome P450-mediated drug metabolism were reviewed in this review without addressing the therapeutic effectiveness of these natural compounds. A promising choice for curing ulcerative colitis are natural plants and products; however, they are not as safe as the public believes and may lead to serious consequences in both short-term and long-term usage. The individual plants are discussed in detail in alphabetical order below.

### 1.1. *Aloe vera*

Aloe vera is a plant that belongs to the Liliaceae family and is extensively used in traditional medicine worldwide. Aloe vera is considered the most widely studied and commercialized plant species. It has been used as a folk remedy for the treatment of a wide range of diseases, including skin problems and gastrointestinal disorders [33,34]. The mucilaginous extract obtained from the meaty leaves has antiinflammatory effects and is used by certain physicians to treat patients with ulcerative colitis [16,34,35]. Several constituents from various phytochemical classes, such as alkaloids, anthrones, chromones, flavonoids, glyocoproteins, naphthalenes and pyrones, have been isolated from different aloe species [36–38]. It is still not yet completely clear which compounds are responsible for the various observed pharmacological properties [35,39,40]. In addition, some components, such as anthraquinones, can have harmful effects, such as genotoxic, mutagenic, and tumour-promoting effects. Further studies need to be conducted to more accurately define the activities of each component [41,42]. Aloe vera is considered a somewhat safe remedy in alternative and complementary medicines (ACM); however, liver toxicity has been reported by several scientists [34,43,44]. Cui and coworkers [45] showed that aloe vera polysaccharide supplementation significantly downregulated the gene expression, protein synthesis and enzyme activities of hepatic CYP1A2 and 3A4 proteins. Similarly, aloe vera juice inhibited the activity of recombinant human CYP3A4 and CYP2D6 enzymes, which are expressed by the metabolism of testosterone and dextromethorphan, respectively [46,47]. There is therapeutic significance to this mechanistic suppression of the CYP3A4 and CYP2D6 enzymes since CYP3A4 and CYP2D6 are the most important enzymes in Phase-I drug responses, accounting for approximately 60%–75% of all phase-I drug reactions [48,49]. In addition, large differences in CYP2D6 metabolism have been observed as a result of genetic polymorphisms in this enzyme. Interference with drug metabolism is indicated by the reported inhibition values, which may have consequences for human drug metabolism as well [46].

It has been observed that aloe vera contains 94.8 mg per kilogram of dry matter of quercetin [50]. Quercetin, a flavanol, reduced the CYP2C9-mediated 4-methylhydroxylation of tolbutamide and the CYP3A4-mediated 6-hydroxylation of testosterone at micromolar concentrations, suggesting that it may interact with conventional medications that are metabolized by CYP2C9 and CYP3A4 [51,52].

Furthermore, anthraquinones are the most important type of components in aloe species, including aloeemodin, rhein, emodin, chrysophanol, physcion and their glucosides [37,53]. Studies have demonstrated that several common herbs, particularly those having high concentrations of anthraquinones and naphthoquinone, have significant inhibitory effects against xenobiotic metabolism. Anthraquinones also exhibit high inhibitory effects against the phase I and phase II metabolism of xenobiotics. The underlying mechanism for this activity might be the strong inhibition of the enzymes CYP1A2, 1A1, and SULT1, which are involved in the metabolism of not only foreign xenobiotics but also endogenous chemicals such as melatonin. Similar inhibitions were further confirmed for recombinant human CYP1A1, 1A2, and 2B1, which were inhibited by emodin with IC50 values of 12.25, 0.67, and 14.89, respectively [54]. More potent inhibitions were also reported [55]. Emodin potently inhibited the activities of recombinant human CYP1A2 (IC50 0.67–7.62 M) and CYP3A4 (IC50 10 M), as well as in human microsomes. More interestingly, this significant inhibition was confirmed in primary human hepatocytes, indicating that it is a general phenomenon [56]. More importantly, recent studies have shown that anthraquinones and their metabolites cause DNA damage [57]. In conclusion, aloe vera, which contains high concentrations of anthraquinones, has the potential to significantly block drug metabolism as well as melatonin metabolism.

Rhein is another anthraquinone metabolite that is abundantly present in aloe vera and other plants [58]. Rhein inhibited the activity of the enzymes CYP1A2, CYP2C9, CYP2D6, CYP2E1, and CYP3A, with Ki values ranging from 10 to 30 μmol in rat liver microsomes, suggesting the possible interaction between rhein and other concomitantly administered drugs [59]. Moreover, aloe vera significantly inhibited the levels of both cytochrome P450 and b5 while inducing cytochrome P450 and b5 reductases in a dose-dependent manner [60]. Thus, aloe vera affects not only CYPs but also the reductase component of the microsomal electron transport system. This observation may be further indicative of interference with oxidative drug metabolism.

In addition, aloe-emodin inhibited the levels of N-acetylation of sulfamethoxazole and p-aminosalicylate, as well as the expression of NAT1 mRNA and protein in A375 cells. S2 cells [61]. Inhibition was further shown to be dose-dependent using the common substrate 2-aminofluorene, which was transformed by NATs to 2-acetylaminoflourene [62]. The findings were validated by the use of a cDNA microarray and a PCR assay to determine gene expression levels. The results demonstrated that aloe-emodin suppresses NAT activity in human melanoma cells by downregulating the production of the NAT gene and protein.

In conclusion, aloe and its phytochemical ingredients have been shown to inhibit all six main CYP families, which are responsible for drug metabolism. Additionally, they seem to block NAT enzymes, which are involved in the metabolism of mesalamine, which is one of the most widely prescribed medications for ulcerative colitis patients. These findings highlight the need for more research on aloe vera, such as hazardous chemical discovery, mechanism-based suppression of CYP activities, and toxicokinetic interactions in both metabolically competent cell and animal models. As a result, aloe is a natural herbal substance that is far from being safe for human use, as suggested in the literature **[34**,^45^,^46^]. When using aloe vera and its products, special attention should be given to the administration of medications, and the advice of a physician should be followed. Otherwise, unavoidable adverse interactions may result as well as catastrophic health consequences.

### 1.2. *Ananas comosus*

Pineapple (*Ananas comosus*) is a tropical plant whose edible fruit is one of the most important tropical fruits in the international trade market [63,64]. Tropical fruits such as pineapple juice and fruit have long been used as traditional treatments to cure digestion system discomforts [65]. In addition to polyphenols and fibres, bromelain is a combination of proteolytic enzymes and the active component found in the pineapple plant A. comosus. Bromelain, a cysteine protease, has been shown to be effective in a variety of therapeutic areas due to its antiinflammatory and anticancer properties, as well as its capacity to promote apoptotic cell death [66]. It possesses antiinflammatory actions and benefits in patients with UC and has been the topic of several investigations [67,68]. The antiinflammatory effect of bromelain is reported to be related to protease activity [69,70]. Bromelain has been shown to be beneficial both clinically and endoscopically in people with mild UC in clinical investigations [64,71].

There are no extensive studies on the drug interactions or interactions with CYPs of pineapple or bromelain. Pineapple fruit juices were implicated in DIs in vitro due to their inhibitory effects on CYP2C9 or CYP3A4. Hidaka and colleagues investigated the effect of bromelain on diclofenac 40-hydroxylase activity, presuming that bromelain is a CYP2C9 inhibitor candidate. At a final dosage of 50 mg/mL, the addition of bromelain resulted in almost total suppression of CYP2C9 activity. The inhibition was concentration dependent, and the IC50 value was found to be 1.2 mg/mL. These findings imply that bromelain is a powerful inhibitor of CYP2C9 and is connected to the inhibitory effect of pineapple juice. CYP2C9 is responsible for the metabolic clearance of several drugs, including warfarin and phenytoin, within a restricted therapeutic plasma concentration range [72]. Pineapple fruit juice, due to its high bromelain content, was found to have the most pronounced inhibitory properties on CYP2C9 compared to other fruit juices. The effect of the inhibition was proportionally dependent on the increase in the amount of pineapple juice. Moreover, starfruit juice was found to be a very potent inhibitor of CYP3A4 compared to grapefruit juice; specifically, an assay of midazolam 1-hydroxylase activity of human CYP3A showed that residual activity of midazolam 1-hydroxylase (%) with starfruit juice was only 0.1 ± 0.0, compared to the 14.7 ± 0.5 for grapefruit juice. It would be interesting to study these inhibitory activities in vivo to determine the clinical relevance of tropical fruit juices on DI [73].

In another study, pineapple juice increased celecoxib and montelukast systemic exposure [74]. Celecoxib is a powerful selective cyclooxygenase-2 (COX-2) inhibitor used to treat inflammatory diseases such as osteoarthritis and rheumatoid arthritis [75]. It is extensively metabolized in the liver, mostly by CYP2C9, and inhibits CYP2D6, although it is not a substrate for this metabolic enzyme [76]. These findings corroborated prior reported findings that pineapple juice had an inhibiting impact on CYP2C9.

However, another study examined the potential protective effects of fruit or vegetable juices against the genotoxicity of heterocyclic aromatic amines (HAAs), which were activated by human xenobiotic-metabolizing enzymes produced in immortal mammalian cells, and the results are very promising. They employed genetically modified metabolically active lung fibroblasts of Chinese hamster V79 cells that included human CYP1A2, NAT2*4 and SULT1A1*1, which were obtained from hamster V79 [77,78]. Despite the fact that pineapple juices provided only marginal protection against HAA genotoxicity, the results provided evidence of the pineapple juices’ inhibitory potential on the CYP1A2 and NAT2*4 enzymes. To better understand these connections, further in-depth research should be conducted.

Thus, the inhibitory impact on CYP2C9 is thought to be the major mechanism underlying pineapple juice interactions, while minor interplay with CYP1A2 and NAT should also be considered. It is reasonable to conclude that pineapple is the safest herbal product to consume among all others; nonetheless, even slight fluctuations in plasma concentrations owing to pineapple interactions may be harmful to individuals taking drugs that are metabolized by the enzyme CYP2C9.

### 1.3. *Andrographis paniculata*

*Andrographis paniculata*, a member of the plant family Acanthaceae, is one of the most prominent medicinal herbs in the world and has been used for centuries in the treatment of a wide range of diseases throughout Asia, America, and Africa. In addition, it contains a number of photochemical compounds with distinct and fascinating biological features. Randomized, double-blinded and multicentre studies investigating the *A. paniculata* extract have suggested similar efficacy to mesalamine for patients with mild to moderate ulcerative colitis [15,79–82].

Studies have reported that *Andrographis paniculata* extract and the major component andrographolide greatly alter both the expression and activity of important P450 isoforms, such as CYP1A1, CYP1A2, CYP2A4, CYP2B9, CYP2B10, CYP2C9, CYP2E1 and CYP3A4, in human hepatocytes [83–90]. There was a time-dependent increase in the mRNA expression of all major CYP isoforms, e.g., CYP1A2, CYP2C9, CYP2E1 and CYP3A4, in human hepatocytes treated with andrographolide. Thus, a risk assessment of A. paniculata should be conducted, since the expression of genes, especially drug metabolism-related genes, might be affected. Moreover, *Andrographis paniculata* extract (APE) inhibited the activities of CYP1A2 and CYP2C in rat and human liver microsomes, but this inhibition was not confirmed in vivo [91–94]. Furthermore, the metabolism of theophylline by CYP1A2 was inhibited significantly with APE [95].

Muthiah and colleagues [96] investigated the effects of several herbs used widely in the southeast, including AP, on the activity of cytochrome P450 2C8 (CYP2C8), an underappreciated human hepatic enzyme involved in drug metabolism. The effects were assessed using amodiaquine N-demethylase activity, which is an in vitro CYP2C8 activity marker. AP had a low IC50 and Ki of 14.8 and 6.26 g/mL, respectively. To the best of our knowledge, this is the first report of AP-induced CYP2C8 suppression. However, the low level of inhibition shown in this investigation suggests that clinically relevant interactions between AP and CYP2C8 substrates are unlikely. The enzyme CYP2C8 is principally responsible for the metabolism of cerivastatin, paclitaxel, rosiglitazone, troglitazone, amodiaquine and chloroquine. Concurrent AP intake may be relevant in drug-herb interactions in vivo through CYP2C8 inhibition. Moreover, interactions between AP and human cytochrome P450 2C19 (CYP2C19), a key hepatic cytochrome P450 isoform implicated in the metabolism of several therapeutic medications, have been studied. Mixed-type inhibition of CYP2C19 was seen in the AP ethanol extract, with moderate Ki values, suggesting that the inhibitors in the extract may bind to both the enzyme and the enzyme–substrate complex, with the same or different affinities [97]. As a result, even if the interaction between AP and CYP2C19 substrates (such as omeprazole, proguanil, barbiturates, citalopram, and diazepam) is minimal, caution should be used when AP is provided concurrently with these drugs.

Aqueous extracts of *Andrographis paniculata* at a concentration of 100 g/mL significantly inhibited CYP3A4 (>85%). This result might be attributed to andrographolide, which had good fitting scores in the docking experiment, as well as the presence of various phenolic compounds in the studied extracts [89]. In addition, by using the fluorogenic CYP assay, *Andrographis paniculata* and its phytochemicals, such as andrographolide, bacoside A, and asiaticoside, were investigated for their ability to inhibit pooled microsomal CYP450s as well as human recombinant CYP3A4, CYP2D6, CYP2C9, and CYP1A2 enzymes, followed by the determination of the inhibitory concentration (IC50). The most potent inhibitory action was shown by A. paniculata against CYP3A4 and CYP2D6 [93,94,98]. Furthermore, both andrographolide and 14-deoxy-11,12-didehydroandrographolide have been shown to inhibit CYP1A2, CYP2D6, and CYP3A4 expression in HepG2 cells [87].

As a result, several different types of CYP isoforms have been observed to interact with AP when tested in vitro. It was discovered that this herb inhibited CYP1A2, CYP2C9, and CYP3A4 more than CYP2D6, CYP2E1, and CYP2B isoforms, indicating that this plant had a distinct inhibitory pattern.

### 1.4. *Artemisia absinthium* [wormwood

Wormwood, or *Artemisia absinthium* L., is an important perennial shrubby medicinal plant native to Asia, the Middle East, Europe, and North Africa [99]. A. absinthium is one of the most significant herbs with a variety of pharmacological properties, including antiinflammatory, antibacterial, antiviral, hypoglycaemic, and hepatoprotective effects, wound healing, and cardiovascular disease prevention [100–104]. Despite concerns about the study’s quality due to nonrandomization, insufficient blinding, the short observational duration, and failure to record adverse effects, randomized controlled studies as well as animal model studies proved the impact of wormwood in IBD [105]. In an open-label study, twenty patients were received either conventional therapy alone or extra *Artemisia absinthium* for six weeks [106]. After 6 weeks, patients in the intervention group had a substantial reduction in disease activity and depression, as well as an improvement in quality of life. Within the control group, no significant changes were discovered. Tumour necrosis factor alpha (TNF-α) levels were also observed to be significantly lower in the intervention group. In another study, patients who were administered wormwood had similar benefits, including reduced intensity of symptoms, enhanced quality of life, decreased depression, and greater well-being [15,107].

Wormwood contains a variety of phytochemicals, including kaempferol, quercetin, and coumarin, which have been demonstrated to inhibit either cytochrome P450 isoform 3A4 or 2B6, which are responsible for the metabolism of its main ingredient artemisinin in humans [104,108,109]. Artemisinin is mostly an antimalarial drug metabolized by CYP2B6 in the liver, with little contribution from CYP3A4 to produce therapeutically inactive metabolites [110]. Furthermore, it was discovered that artemisinin suppressed human microsomal CYP2B6 activity but not CYP3A4 activity [111]. Furthermore, Xing and colleagues [112] demonstrated that artemisinin medicines are possible inducers of P450 enzymes, with the most inducible enzymes being CYP2B6 and CYP3A4, which are thought to be the key enzymes implicated in artemisinin drug autoinduction. Artemisinin drugs also exhibited mild inhibition of CYP2B6 but not CYP3A4. In addition, artemisinin was reported to inhibit CYP2B6 in vitro through a partly mixed manner of inhibition in another investigation. Artemisinin’s mechanism was neither time-dependent nor NADPH-dependent, suggesting a reversible manner of inhibition. However, the projected degree of CYP2B6 inhibition in vivo by both artemether and artemisinin was suggested to be moderate [113]. Thus, wormwood may endanger conventional medications processed by CYP2B6 and CYP3A while also enhancing artemisinin bioavailability.

Eupatilin and jaceosidin are pharmacologically active flavones discovered in Artemisia species. Although they are not reported in A. absinthium, similar methoxylated flavones have been reported [114,115]. Eupatilin and jaceosidin Eupatilin and jaceosidin were reported to inhibit CYP1A2-catalysed phenacetin O-deethylation with IC50 values of 9.4 μM and 5.3 μM, respectively, as well as CYP2C9-catalysed diclofenac 4-hydroxylation with IC50 values of 4.1 μM and 10.2 μM, respectively. They were also shown to have a considerable inhibitory effect on CYP2C19-catalysed [S-mephenytoin 4′-hydroxylation, CYP2D6-catalysed bufuralol 1′-hydroxylation, and CYP2C8-catalysed amodiaquine N-deethylation. A kinetic study of human liver microsomes suggested that eupatilin and jaceosidin are competitive inhibitors of CYP1A2 and a mixed-type inhibitor of CYP2C9 [116]. At the highest dose tested (100 μM), eupastilin and jaceosidin demonstrated no inhibition of CYP2A6-catalysed coumarin 7-hydroxylation and CYP3A4-catalysed midazolam 1′-hydroxylation. These in vitro findings imply that wormwood should be investigated further for possible pharmacokinetic drug interactions in vivo owing to CYP1A2 and CYP2C9 suppression.

The modulation of cytochrome P450 activities of Artemisia plant extracts (water or ethanolic) on human cells, as well as the antiinflammatory activity, have been researched by pioneering researchers [117,118]. There was a substantial decrease in CYP3A4 activity in all Artemisia plant samples (*A. annua*, *A. afra*, *A. abrotanum*, *A. absintium*, *A. apiacea*, *A. herba alba*, and *A. pontica*) tested. The amount of CYP3A4 inhibition observed with all Artemisia samples was unexpectedly large, reaching up to 6 times the levels observed in ketoconazol or diluted grapefruit juice. Because grapefruit juice has a reputation for being the most powerful CYP3A4 inhibitor derived from a plant, this is shocking to learn that all Artemisia samples exhibit this high level of CYP3A4 inhibition.

In conclusion, the DDI prediction indicated that Artemisia might result in both induction and inhibition. Additionally, combination medicines (including artemisinin) cleared by CYP2B6 and CYP3A4 and, to a lesser extent, CYP1A2 and CYP2C9 may need a dosage adjustment to sustain treatment or prevent adverse effects. In addition, considering its well-known potential toxic effects [119] highlighted by the known neurotoxicity of thujones, its utilization should be restricted.

### 1.5. *Boswellia serrata*

The *Boswellia serrata* tree is a moderately large deciduous tree that is native to India, the Middle East, and Northern Africa. When the paper-thin bark of the Boswellia tree is peeled back, sticky oleoresin is revealed. Extracts of this oleoresin have been utilized in Ayurvedic medicine to treat inflammatory diseases and gastrointestinal issues for its antiinflammatory, antiseptic, astringent, and stimulating action [120–122]. Studies have shown that patients with chronic colitis who were treated with Boswellia gum resin showed decreased intensity of symptoms and better quality of life [123,124]. Clinical, haematological and biochemical parameters exhibited declining trends with complete resolution of ulcers after 6 weeks of treatment [120,121,125,126].

A type of Ayurvedic medicine, BV-9238, a proprietary formulation of *Withania somnifera*, *Boswellia serrata*, *Zingiber officinale*, and *Curcuma longa*, was investigated for its toxic effects and cytochrome P450 enzyme inhibition activity [127. Human recombinant CYP1A2, CYP2C19, CYP2C9, CYP2D6, CYP2E1, and CYP3A4 isomers were tested using 3-cyano-7-ethoxycoumarin as a substrate, while the CYP3A4 isomer employed 7-benzyloxy-4-(trifluoromethyl)-coumarin. Although no particular prototype substrate was used at different doses, CYP2C9 and CYP3A4 were the most susceptible to inhibition, with 35% and 19% inhibition, respectively, at a concentration of 0.5 g/mL of BV-9238, while CYP4502E1, CYP450 2D6, and CYP450 2C9 activities were the least sensitive. Similarly, total boswellic acids were shown to be effective inhibitors, while additional components in *Boswellia serrata* extract play an important role [127].

The inhibition of the applied CYP enzymes 1A2/2C8/2C9/2C19/2D6 and 3A4 by the frankincense extracts (*Boswellia carteri*, *Boswellia frereana*, *Boswellia sacra* and *Boswellia serrata*) was determined using LC/LC/ESI-MS. The boswellic acids 11-keto-β-boswellic acid and acetyl-11-keto-β-boswellic acid showed the highest inhibitory activities for the CYP enzymes 2C8/2C9 and 3A4, with IC50 values in the range of 5 to 10 μM. Since the gum resin of B. frereana also showed a comparable strong inhibition of the applied CYP enzymes, the boswellic acids are obviously not primarily responsible for the potent inhibitory activity of the frankincense extracts. Therefore, phytoconstituents other than boswellic acid and its metabolites exert P450 inhibitory actions. Since herbal extracts of B. serrata are widely used for the treatment of inflammatory diseases, further in vitro and in vivo studies are needed to clarify the clinical relevance of these findings [128].

The authors provided case reports of suppression of metabolic pathways (CYP2C19, CYP3A4, and CYP2C9) [129]. Incidences of an interaction between the herb *Boswellia serrata* and the anticoagulant warfarin have been recorded. Because it inhibits lipoxygenase and interacts with COX-1, it is possible that boswellic acid is the source of the interaction between boswellia and warfarin. Furthermore, boswellia has been shown to inhibit the isoenzymes CYP2C19, CYP3A4 and CYP2C9, which are responsible for the metabolism of warfarin, hence increasing its anticoagulant efficacy [122,130,131]. According to current research, the use of boswellia in patients who have achieved warfarin stabilization is not recommended. Given the estimated number of prescriptions (14,632,370) in the United States in 2019, the boswellic acid interaction is highly critical. Furthermore, *Boswellia serrata* extract as well as boswellic acids, 11-keto-β-boswellic acid, and acetyl-11-keto-β-boswellic were identified as an additional class of potent P-glycoprotein (Pgp) inhibitors based on the modulation of Pgp-mediated efflux. Thus, it also affects drug transport in the gastrointestinal tract as well as drug metabolism [132].

It became apparent that Boswellia extracts and frankincense contain several phytochemicals that modulate CYP2C19, CYP3A4 and CYP2C9 as well as Pgp, suggesting unavoidable adverse interactions that may pose serious health consequences. It is obvious that more research is needed to obtain definitive conclusions about Boswellia toxicities in the future.

### 1.6. *Cannabis sativa*

*Cannabis sativa*, usually known as Indian hemp, is an herbaceous annual plant that has been grown mostly in Central Asia (India and China) since antiquity [133]. C. sativa has been used for millennia as a source of fibre, food, oil, and medicine, as well as for recreational and religious purposes [134]. It includes many chemically active chemicals, including cannabinoids, terpenoids, flavonoids, and alkaloids [135,136]. Δ9-Tetrahydrocannabinol (THC) and cannabidiol (CBD) are the most important phytocannabinoids. Clinical investigations have shown that cannabis has beneficial effects in the gut, as well as the capacity to decrease inflammation, discomfort, and hypermotility, and that it may be utilized in the treatment of ulcerative colitis [124,137–143].

Numerous in vitro and in vivo investigations suggest that cannabis may impact P450 isoenzymes to alter drug metabolism. The comprehensive analysis implicated the role of P450 in the metabolism of various exogenous cannabinoids, including tetrahydrocannabinol (THC; CYP2C9, 2J2; 3A4), cannabidiol (CBD; CYP2C19, 2J2; 3A4), and cannabinol (CBN; CYP2C9, 2J2, 3A4) [144–148]. Cannabinoid inhibition or induction of CYP (e.g., THC as a CYP1A2 inducer and CBD as a 3A4 inhibitor) may influence the metabolism of various medications metabolized by these CYPs [136,144,148–153]. However, the applicability of experimental findings in cells or animals to humans has yet to be proven, and specific clinical trials are required to validate these interactions. The effects of cannabis and its major phytoconstituents are listed as follows:

Cannabis, particularly CBD, has a strong inhibitory effect on CYP1A2 activity. Competitive inhibition of 7-ethoxyresorufin O-deethylase activity catalysed by recombinant hCYP1A2 was observed in the presence of these cannabinoids. CBD was shown to be the most effective inhibitor of CYP1A activity. CBN was also efficient at decreasing the activity of CYP1A2. However, THC was a less powerful inhibitor of CYP1 activity and less selective against CYP1 inhibition than CBD and CBN [154]. In contrast, marijuana inhalation increased the expression of the CYP1A2 enzyme [155]. Thus, the route of administration of cannabis is important in exerting selective actions on CYP1A family members. In addition, it was discovered that caffeine metabolism mediated by CYP1A2 was minimally affected in the presence of cannabinoids [153].

THC, CBN, and CBD were found to be highly effective inhibitors of the CYP2A6 and CYP2C9 enzymes but a weaker inhibitor of CYP2B6. THC and CBN both displayed mechanism-based inhibition of the enzyme CYP2A6. The cannabinoids THC, CBD, and CBN suppressed the coumarin 7-hydroxylase activity of recombinant CYP2A6 in a noncompetitive manner, with apparent Ki at approximately 30–40 μM. THC, CBD, and CBN inhibited recombinant CYP2B6 7-dependent benzoxyresorufin O-debenzylase activity in a mixed fashion in a dose-dependent manner, with very small Ki values at approximately 2 μM, suggesting stronger inhibitory action on CYP2B6 than CYP2A6 [156]. Similarly, another study demonstrated that the majority of the cannabinoids tested were shown to decrease CYP2B6 activity to a limited extent [153]. In the presence of THC, CBN, CBD, and CBC, the apparent Vmax of CYP2J2-dependent AEA metabolism was lowered to 20%–45% of that of the uninhibited control enzyme [145].

A competitive inhibition study of CYP3A revealed that THC, CBD, and CBN were all effective inhibitors, with CBD being the most potent at a concentration comparable to that found in typical cannabis inhalation. CBD was shown to be the most powerful inhibitor of CYP3A4 and CYP3A5 among the three main cannabinoids tested, and it was found to be competitive. The IC50 values for THC or CBN for CYP3A4 or CYP3A5 were quite high. In addition, CYP3A7 activity was suppressed to a comparable level by these cannabinoids but in a mixed manner [155,157]. Moreover, CBD competitively prevented the synthesis of 2 alpha-hydroxy-testosterone and 16 alpha-hydroxy-testosterone, which was catalysed by a reconstituted system containing purified hepatic cytochrome P-450. However, it had no effect on the production of androstenedione (androst-4-ene-3,17-dione) or 7 alpha-OH-testosterone. According to the results of the kinetic analyses, CBD had a distinct inhibitory profile for testosterone oxidation in comparison to the profile of SKF 525-A. However, partial suppression of the CYP3A4-dependent metabolism of nifedipine and testosterone has been observed in certain studies [153].

CYP2C9 was found to be inhibited by major cannabinoids, with the inhibitory effect of CBD being dependent on the substrates used in the experiments. Most of the cannabinoids reduced the CYP2C9-mediated metabolism of tolbutamide by more than 50%, and as a result, they were further investigated. The inhibitory concentrations (IC50) of CBD, CBDV, cannabigerolic acid and THCA varied between 2.5 and 6.4 μM [153]. Cannabinol (CBN) was the only cannabinoid shown to have no effect on the activity of the CYP2C9 enzyme. Hepatocytes and recombinant CYP2C19 were shown to have decreased (S)-mephenytoin 4′-hydroxylase activity when exposed to CBD in a concentration-dependent mixed manner. Additionally, CBD reduced the activities of recombinant CYP2C19, which catalysed omeprazole 5-hydroxylase and 3-O-methylfluorescein O-demethylase [153,155,158–160]. It is possible that cannabis usage will inhibit CYP2C9 to a degree that is clinically meaningful for people on warfarin, a very common drug used to reduce the risk of clotting [147]. Thus, it is highly recommending that such practices be strictly prohibited and that clinical drug-drug interaction research be conducted to determine the impact of marijuana on CYP2C9 activity. Similar to CYP2C9, THC, CBD, and CBN were found to inhibit CYP2D6, with CBD being the most potent when administered at a higher concentration than that found in typical cannabis consumption [146]. All of these major cannabinoids inhibited the activities of recombinant CYP2D6 and pooled human liver microsomes in a concentration-dependent manner, with CBD demonstrating the greatest inhibitory potency [161]. However, it has been shown that dextromethorphan O-demethylase activities are only partially inhibited [153].

In summary, the effects of cannabis and its phytoconstituents on CYPs are undeniable. There is enough evidence to draw firm judgement on the safety of cannabis or cannabidiol in people with UC. To obtain clearer findings on the safety of cannabis usage, studies with greater methodological quality and a larger number of participants with long-term follow-up are necessary. Patients should be told that the use of cannabis and cannabinoids may result in DDIs that have an impact on the effectiveness, tolerability, and safety of their medication.

### 1.7. *Curcuma longa*

Turmeric (*Curcuma longa* L.) is a perennial herb that is a member of the Zingiberaceae family of plants. It is one of the most important Indian spices and natural colouring agents due to its rhizomes. From ancient times to the present, this plant has been widely used for a variety of purposes, including for therapeutic purposes in foods [162–166]. Turmeric is also one of the most popular dietary supplements in the world [167]. Curcuma spp. has yielded over 427 chemical compounds, all of which have been isolated and identified. This genus has a high concentration of flavonoids, tannins, anthocyanin, phenolic compounds, oil, organic acids, and inorganic compounds, among other things. Curcumin, one of the most important active components in Curcuma, is known for its powerful antiinflammatory and antioxidant properties. Furthermore, pharmacological research has shown that Curcuma has a broad variety of actions, including hepatoprotective, antifungal, antihypertensive, and neuroprotective properties [162, 168–174]. It has been reported that curcumin-loaded nanocarriers significantly improve CUR’s antiinflammatory properties, enhancing its overall therapeutic efficacy [175]. Curcumin supplementation demonstrated some additional therapeutic efficacy in patients with UC when taken in conjunction with antiinflammatory medicines [176–179]. Conversely, conflicting findings indicated that low-dose oral curcumin was unsuccessful in producing remission in mild to moderate instances of UC [180]. Thus, curcumin seems to be a potential therapy for sustaining remission in people with quiescent, mild to moderate UC, but further research on curcumin is needed to support the present results.

Curcumin is a competitive inhibitor of CYP1A2 with an IC50 ranging from 40–100 μM according to in vitro testing involving human CYP enzymes produced in the *E. col*i cell membrane or liver microsomes (Appiah-Opong et al., 2007; Bamba et al., 2011). Likewise, other curcuminoids were found to inhibit CYP1A2, which accounts for approximately 13% of human liver cytochromes, and biotransform arylamine drugs such as theophylline and lidocaine [127,181–186]. However, human liver CYP1A2-dependent phenacetin O-deethylation activity has been reported to remain unaltered with curcumonol [187].

Curcumin at concentrations ranging from 0.9 to 100 μM was shown to be a competitive inhibitor of the CYP2B6 enzyme, a member of the CYP2 family that plays an important role in chemotherapeutic drug metabolism. For example, cyclophosphamide [181,182,188–191] Curcuma extract reduced CYP2C19 activity as a noncompetitive inhibitor with an IC50 of 4.3−7.4 μM; CYP2C19 is an isoform involved in the biotransformation of approximately 20% of the medications on the market [183,187,189,192–195].

In human epithelial colorectal adenocarcinoma cells, turmeric extracts were found to inhibit the activity of the intestinal CYP3A4 isoform by approximately 50% [196]. Pure curcumin also induced 30%–40% inhibition of the CYP3A4 isoenzyme in Caco-2 cells, similar to the results obtained with curcumin extract [189,196,197]. Curcumin was shown to be a competitive inhibitor of the CYP3A4 isoform. In addition, other in vitro investigations of CYP isoforms have revealed the inhibitory effects of curcumin and other curcuminoids [127 184,186,187,190,192,198,199]. Several additional studies utilizing intestinal human colon cancer cell lines, human hepatocytes, and HepG2 cells likewise failed to find any significant effects of curcumin on the mRNA expression of CYP3A4 [181,200–202]. The CYP3A4 isoform is the most notable CYP in that it is the primary drug-metabolizing enzyme in liver and intestinal tissue, catalysing the metabolism of half of all commercially available pharmaceuticals [203–205]. In addition to its significant role in first-pass metabolism, it plays a significant role in the metabolism of endogenous substrates such as cholesterol and bile acids [206,207]. Thus, well-designed clinical studies in healthy volunteers as well as patients are required to obtain an acceptable level of evidence required to establish curcumin-CYP3A4 interactions in humans.

The effect of curcumonoids on CYP enzymes (e.g., CYP17A1, CYP19A1, and CYP21A2) involved in the steroidogenic pathway was also investigated in another research study. Both the 17-hydroxylase and 17,20-lyase activities of CYP17A1 and the aromatase activity of CYP19A1 were considerably inhibited [191,208]. Analogously, curcumin extract was shown to strongly inhibit 5aR1, an enzyme implicated in the NADPH-dependent reduction of steroids [209]. Therefore, although they are not involved in drug metabolism, studies have shown that curcuma and curcuminoids may induce suppression of steroid metabolism as well as perturbation of the related processes. A critical concern in the use of turmeric is the inaccurate categorization of Curcuma species, which occurs as a result of the large number of closely related species. Because of this, Curcuma species (Zingiberaceae) have been misclassified, resulting in unintended human exposure to phytochemicals that alter CYP expression and activity [210]. Additionally, it was reported that NAT activity in human A549 cells and cytosols was suppressed by curcumin in a dose-dependent manner; NAT is responsible for the metabolism of 5-ASA to inactive metabolites. The results also demonstrated that NAT1 mRNA expression was inhibited and decreased by curcumin.

As a result, curcuma and its main constituent curcumin interact with the drug-metabolizing enzymes CYP1A2, 2B6, 2C9 and 3A4, NAT, CYP17A1 and 19A1. It affects the pharmacokinetics of medications taken concurrently in conjunction with it, despite its general public safety perception.

### 1.8. Germinated barley foodstuff/wheat grass juice

Germinated barley foodstuff (GBF), which is composed of dietary fibre and glutamine-rich protein that serves as a probiotic, has been shown to lessen the recurrence rate and clinical reduction in disease activity in patients [211–214]. On the basis of its high water-holding capacity and ability to control microbiota, it has been hypothesized that GBF may play a significant role in the prolongation of remission and therapy in UC [16,212,215–217]. Similarly, administration of wheat grass juice resulted in considerably reduced disease activity, decreased rectal bleeding, and decreased stomach discomfort. Because this inquiry was intended to be a pilot study, the findings may serve as a foundation for more clinical studies in the future with larger sample numbers, healthy control groups, and higher dosages of GBF and WGJ [15,218].

There have been no reported interactions between Hordeum vulgare and Triticum aestivum with any of the P450 isoforms that are involved in the metabolism of drugs. As a result, they are considered to be the safest alternatives to conventional drugs for the treatment of UC.

### 1.9. *Indigo naturalis*

*Indigo naturalis* (IN) is an herbal medication made from the leaves and stems of plants such as *Indigofera tinctoria*, *Strobilanthes cusia*, and *Polygonum tinctorium*. It is used to treat a variety of diseases, including UC. IN is an herbal medicine that is quality controlled in China if it contains more than 2% indigo and 0.13% indirubin, which is the legal limit. IN that originates in Fujian is often considered to be of the best quality. In randomized, placebo-controlled clinical trials, IN was shown to be useful in the treatment of ulcerative colitis [219–224]. Unfortunately, even though there is evidence that IN may be beneficial in the treatment of UC, there are many unknowns, including the effective components and therapeutic targets that remain to be discovered. A recent study applied a network pharmacology method to anticipate the molecular mechanism that underlies the action and the effective components of IN in the treatment of UC in general [225].

There are numerous unknowns about both the efficacy of IN for UC and the interaction of IN with drug metabolizing enzymes. The data that are currently available, even if limited, indicate that IN may offer a considerable threat when used in conjunction with currently utilized pharmaceuticals [224,226]. CYP1A1 expression in colon tissue from six patients was found to have increased by a factor of 12.557 after 8 weeks of IN treatment [224]. However, although CYP1A1 is involved in the metabolism of a few drugs, such as theophylline, it is mostly engaged in the metabolic activation of aromatic hydrocarbons into potentially carcinogenic compounds [227,228]. Furthermore, both indigo and indirubin were discovered to be AhR ligands, implying that the expression of genes controlled by AhR, including CYP1A2, is changed [229–231]. In addition, indirubin activated the transcription of the CYP3A4 gene in HepG2 cells via the activation of the PXR gene. More in-depth investigations into the molecular mechanism of indirubin-induced CYP3A4 activation may provide vital insights into the interactions between herbs and drugs as well as drug-drug interactions [232]. Another study sought to determine the possible effects of Realgar-Indigo naturalis (RIN) on the activities of four CYP450 isozymes, specifically the activities of CYP1A2, CYP2C11, CYP2E1, and CYP3A1/2 in rats. The findings revealed that RIN has the ability to considerably suppress CYP1A2 enzyme activity while simultaneously increasing CYP2C11 enzyme activity. Unexpectedly, the RIN high dosage group showed considerable inhibition of CYP3A1/2 enzyme activity, with various dosages exhibiting a good dose-dependent response [233].

Thus, all these limited findings of IN suggested that drugs administered in conjunction with IN may need special consideration in terms of drug-drug interactions, and further detailed studies are required.

### 1.10. Miscellaneous

A number of other herbal products exhibiting antiinflammatory properties are reportedly used in the treatment of UC, and various preclinical and clinical studies are currently available. Among the most notable plants that have been utilized are *Bletilla striata*, *Capparis ovata*, *Commiphora wightii*, *Cynara scolymus*, *Fumaria officinalis*, *Glycyrrhiza glabra*, *Glycyrrhiza uralensis*, *Hypericum perforatum*, *Mentha piperita*, *Momordica charantia*, *Oenothera biennis*, *Plantago ovata*, *Plantago psyllium*, *Potentilla erecta*, *Sanguisorba officinalis*, *Sophorae flavescentis*, *Sorbus domestica*, *Strobilanthes cusia*, and *Withania somnifera* [13,15,16,220,234–251]. However, due to methodological flaws, heterogeneity in the definition of diagnostic criteria, and the absence of placebo control and blinded measurement of subjective conclusions, the majority of trials associating herbal medicines with conventional therapies have not provided significant evidence to support the use of herbal medicines. In addition, it is unfortunate that there are no detailed studies on the occurrence of acute or chronic side effects and an understanding of more subtle and potentially long-term consequences involving DME interactions. There is a paucity of credible evidence on the effectiveness and safety of the majority of these plants in UC patients. Therefore, the exact mechanisms of action of these herbal products and their interaction with the various cytochrome P-450 isoforms remain a mystery at this time.

## 2. Conclusion

The most notable characteristics of traditional herbal medicines are undoubtedly their multicomponent and multieffect natures. Overall, the clinical relevance of the CYP system is well known in drug-drug interactions as well as herbal medicine-drug interactions because various xenobiotics, including those found in traditional herbal medicines, can influence their activity and thus have an impact on systemic drug exposure, i.e. efficacy and toxicity. As shown by the studies examined in this review, herbal medicines used to treat ulcerative colitis are not exempt from this phenomenon (Table).

In conclusion, despite their possible antiinflammatory properties, herbal products should not be prescribed due to their interactions with drug-metabolizing enzymes and adverse events, according to the canonical primum nonnocere (first, do no harm) medical concern. Moreover, considering the polymorphisms in human CYP genes, which largely alter the function of the enzymes, the prediction of adverse effects is almost impossible. Thus, physicians should encourage their patients to declare their use of complementary medicines to avoid significant consequences that may result from concurrent use of conventional pharmaceuticals.

## Figures and Tables

**Table:** An overview how the herbal products used to treat ulcerative colitis affect major drug-metabolising enzymes.

Herbal Product	Major drug metabolising CYPs						Others			
	**1A2**	**2B6**	**2C9**	**2C19**	**2D6**	**3A4**	**1A1**	**2B1/9**	**2E1**	**NAT**
Aloe vera	↓↓↓	−	↓↓↓	−	↓↓	↓↓↓	↓↓	↓	↓↓↓	↓↓
Ananas comosus	↓	−	↓↓↓	−	−	↓↓↓	−	−	−	↓
Andrographis paniculata	↓↓↓	−	↓↓	↓↓	↓	↓↓↓	↓	↓	↓↓	−
Artemisia absinthium	↓	↓↓↓	↓	↓↓	↓↓	↓↓↓	−	−	−	−
Boswellia serrata	−	−	↓↓	↓↓	−	↓↓	−	−	−	−
Cannabis sativa	↑/↓↓	↓	↓↓↓	↓↓	↓↓	↓↓↓	−	−	−	−
Curcuma longa	↓↓	↓↓	−	↓↓↓	−	↓↓↓	−	−	−	↓↓↓
GBF/WGJ	−	−	−	−	−	−	−	−	−	−
Indigo naturalis	↓	−	−	−	−	↑↑/↓↓↓	↑↑↑↑	−	↑	−

−: no data, one-arrow: weak, two-arrow: moderate; three-arrow: strong; ↑: increase; ↓: decrease.
